# Label-Free Quantification by Liquid Chromatography–Tandem Mass Spectrometry of the Kunitz Inhibitor of Trypsin KTI3 in Soy Products

**DOI:** 10.1021/acs.jafc.3c01173

**Published:** 2023-05-23

**Authors:** Barbara Prandi, Chiara Vacca, Stefano Sforza, Tullia Tedeschi

**Affiliations:** Department of Food and Drug, University of Parma, parco area delle scienze 17/A, Parma 43124, Italy

**Keywords:** antinutritional factor, trypsin inhibitor, KTI3, soy, LC-MS, marker peptide

## Abstract

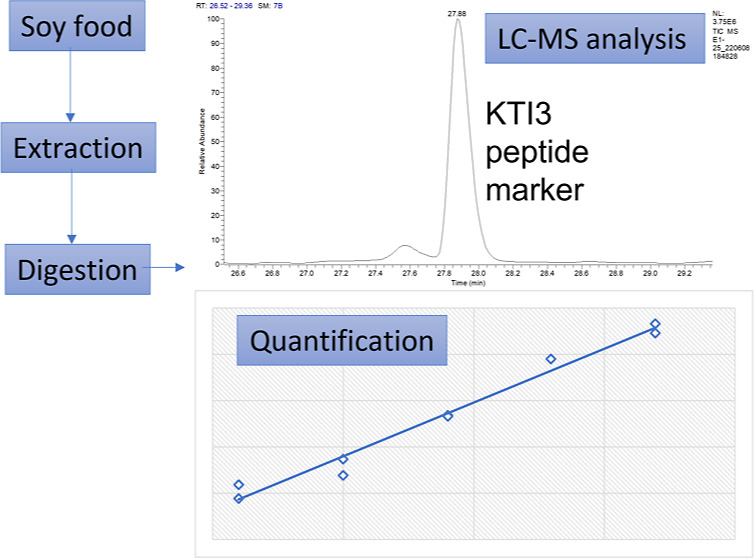

The greater awareness of consumers regarding the sustainability of food chains has shifted part of the consumption from animal protein sources to vegetable sources. Among these, of relevance both for human food use and for animal feed, is soy. However, its high protein content is unfortunately accompanied by the presence of antinutritional factors, including Kunitz’s trypsin inhibitor (KTI). Now there are few analytical methods available for its direct quantification, as the inhibitory activity against trypsin is generically measured, which however can be given by many other molecules and undergo numerous interferences. Therefore, in this work, a direct label-free liquid chromatography–mass spectrometry (LC–MS) method for the identification and quantification of trypsin Kunitz inhibitor KTI3 in soybean and derivative products has been developed. The method is based on the identification and quantification of a marker peptide, specific for the protein of interest. Quantification is achieved with an external calibration curve in the matrix, and the limit of detection and the limit of quantification of the method are 0.75 and 2.51 μg/g, respectively. The results of the LC–MS method were also compared with trypsin inhibition measured spectrophotometrically, highlighting the complementarity of these two different pieces of information.

## Introduction

The demand for soybean-based ingredients is continuously growing due to their inclusion in a multitude of food and feed products: soybean oil for shortening, soy flour and protein in feed production, and vegetable protein substitutes for meat and dairy products.^1^ Despite their widespread and ever-increasing use, soy-derived ingredients present several problems that can affect their nutritional and biological properties: phytoestrogens (potent activators of estrogen receptors that induce biological effects similar to endogenous and synthetic estrogens),^2^ allergens (about 0.2–0.4% of people allergic to soy),^3^ possible presence of mycotoxins and/or alkaloids^4^ (which can pose a significant health risk), and antinutritional compounds (acting through several mechanisms, including enzyme inhibition).^5^

Antinutritional factors are naturally occurring compounds in various foods which, as the name suggest, can impair the nutritional quality of those foods. Among the best known antinutritional factors are glucosinolates in mustard and rapeseed protein products, trypsin inhibitors (TIs) and hemagglutinins in legumes, tannins in legumes and cereals, gossypol in protein products cottonseed, and uricogenic nucleobases in yeast protein products.^6^ Kunitz trypsin inhibitor (KTI) and agglutinins (belonging to the protein class), phytic acid and oligosaccharides of the raffinose family (belonging to the carbohydrate class) are the main antinutritional factors of soybean.^7^ The presence of phytic acid negatively affects the digestibility of proteins and reduces the bioavailability of minerals.^8^ Large amounts of galacto-oligosaccharides such as raffinose and stachyose, which are indigestible by mammals, are factors in causing flatulence.^9^ Soy agglutinin is responsible for the hemagglutinating activity of soybeans, directed toward erythrocytes and other cells as well.^10^

Trypsin inhibitors (TIs) are among the most relevant antinutritional factors because they reduce the digestion and absorption of dietary proteins by inhibiting the digestive enzyme trypsin.^11^ Protease inhibitors are in fact widely distributed in plants to play a protective role against herbivores: their action consists in inhibiting digestive proteolytic enzymes, in particular trypsin and chymotrypsin, blocking their enzymatic activity. Therefore, when ingested together with a protein source, a reduced digestibility and bioavailability of proteins is observed.^12^ As for soybeans, the seeds contain about 35–40% protein, and TIs make up about 6% of their protein. Although their inhibitory activity is largely inactivated by the heat treatments conventionally applied to soybean meal, still 10–20% of residual activity remains in the final ingredients.^13^ One of the most studied and characterized TI in soy is the Kunitz inhibitor. The Kunitz inhibitor is a small protein having a molecular weight of about 21.5 kDa, consisting of a single polypeptide chain cross-linked by two disulfide bridges.^14^ The Kunitz inhibitor works by forming a 1:1 stoichiometric complex with the protease active site, which in turns cleaves a single arginine–isoleucine bond on the inhibitor. The inhibition is reversible and pH-dependent^15^ and, given the mechanism, the amount of inhibition is strictly related to the amount of the inhibitor.^16^ The effect of TIs would not only be a consequence of the inhibition of intestinal digestion but also of an enlargement of the pancreas (hypertrophy and hyperplasia, observed in rodents and birds) and a hypersecretion of digestive enzymes. The loss of endogenous sulfur-rich proteins (trypsin and chymotrypsin) would lead to growth inhibition, also considering that soy proteins are deficient in these amino acids.^17^ Several research studies have been targeted to the development of Kunitz-free soybean genotypes^18^ and how to identify them.^19^

In parallel, much interest has been devoted to the different ways of inactivating the inhibition. As already said above, heat treatments are among the most effective ways to avoid the inhibitory effect. However, heat treatments are expensive, require a lot of energy, can influence the structure of proteins, and therefore modify their functionality for certain food applications.^20,21^ The kinetics of inactivation of TIs by heat is a two-phase process^22^ and, among the different treatments, boiling, autoclaving, and microwave irradiation (especially when coupled with soaking) significantly reduce Kunitz inhibitor in soy, as determined by native polyacrylamide gel electrophoresis (PAGE) and Western blotting. More specifically, in that study, boiling and autoclaving for 15 min both resulted in complete inactivation of the Kunitz inhibitor, whereas microwave irradiation induced a significantly higher reduction for the Kunitz inhibitor in soaked versus dry seeds.^23^ The inactivation of Kunitz inhibitor depends on temperature, time, *a*_w_, and matrix: purified Kunitz inhibitor lost most of its activity after 180 min of heat treatment (about 20% of residual activity), and soybean extract after only 30 min (almost no residual activity). The inactivation of the Kunitz inhibitor is faster at *a*_w_ 0.75 (about 30% residual activity after 150 min) than at 0.50 and 0.32 (almost no inactivation), and higher at 95 °C (about 30% residual activity after 150 min) than at 85 °C (about 75%) and at 75 °C (almost no inactivation).^24^ In another study, Kunitz inhibitor was shown to lose its activity after 20 min at 120 °C, while about half of its activity was maintained at 100 °C (in 0.05 M TrisHCl buffer pH 8).^25^ With the heat treatment, therefore, the inhibitor decreases its activity, until it is completely (or almost) deactivated under certain conditions. The protein therefore remains present and potentially detectable by direct methods, while it is no longer detected by indirect methods which are based on its inhibitory activity.

The quantification of TIs is necessary because they influence the nutritional properties of the foods in which they are contained. Immunoassays based on the enzyme-linked immunosorbent assay (ELISA) technique have been largely used for this purpose; among these, the sandwich ELISA has an approximately 5 times greater sensitivity for the Kunitz-type inhibitor than the competitive ELISA.^26^ Spectrophotometric methods are also largely diffused to indirectly quantify TIs by measuring the inhibition of a trypsin standard solution. An example is the official British standard BS EN ISO 14902:2001: TIs are extracted from the sample at pH 9.5 and trypsin activity is measured by adding benzoyl-l-arginine-*p*-nitroanilide (L-BAPA) as the substrate. The amount of *p*-nitroaniline released is then measured spectrophotometrically.^27^ This method has some limitations: it requires numerous attempts before identifying the right dilution of the sample to be in the range of linearity of the response (40–60% inhibition), some of the reagents must be prepared fresh daily, as well as the extracts deriving from the samples (which therefore must be re-extracted if the analysis cannot be completed within the day). Finally, being an indirect method, it determines any substance or condition that inhibits trypsin activity, so it is not specific for the Kunitz inhibitor. A two-dimensional liquid chromatography method was developed to quantify Kunitz-type inhibitor in soybeans. This method first involves the use of ion exchange chromatography to collect the fractions of the soybean extract; then, the fraction containing the Kunitz inhibitor is further resolved by size-exclusion chromatography with diode array detection (DAD), which is also used for the quantification of the Kunitz-type inhibitor. The amounts of KTI in the soybean samples were determined using a calibration curve constructed with the KTI standard solution, with a limit of detection of 0.12 mg/g.^28^ Another reverse-phase liquid chromatography method with UV detection at 220 nm was also developed for the quantification of Kunitz-type inhibitor in soybeans. Quantification was performed using an external calibration curve made with KTI standards, with a detection limit of 0.05 mg/g.^29^

To overcome the requirement of complicated multiple steps for the analysis of intact whole proteins in complex matrices, proteins can be cleaved with specific proteases into shorter peptides, which pose less analytical problems than whole proteins. The identification of one (or more) peptide marker within the target protein sequence allows its easy quantification by liquid chromatography–mass spectrometry (LC–MS) techniques. This approach has already been applied, also by our group, for α-amylase/TIs in wheat^30−32^ with good results. For what concerns soybean, LC–MS methods have been developed to quantify the alpha subunit of conglycinin (detection limit of the marker peptide of 0.48 ng/mL)^33^ and lectin (another major antinutritional factor in soy), with a detection limit of 35.5 μg/g.^34^ In the field of allergen quantitation, isotopically labeled peptides have been used to quantify the content of 10 allergenic proteins in soybean.^35−37^ KTI3 was present in amounts ranging from 1.0 to 4.2 μg peptide/mg protein. Limits of detection and quantification have not been provided for these methods.

In the present work, UHPLC/ESI-MS/MS has been developed to quantify the Kunitz type inhibitor KTI3 in different soy products using a proteo-typic marker peptide following enzymatic digestion. Among the different isoforms of the Kunitz inhibitor, KTI3 was chosen because the KTI3 gene encodes the predominant TI in soybeans.^38^ The high selectivity of MS can avoid long and/or multi-step chromatographic separations, and being specific for a certain peptide, interference of co-extracted compounds which absorb at 220 nm in UV detection is eliminated. Furthermore, since the use of isotopically labeled peptides can be expensive and standard Kunitz inhibitors are commercially available, the method developed here involves quantification via an external calibration curve made with the commercially available KTI standard. The standard (therefore the intact KTI protein) is subjected to the same extraction and digestion procedure as the samples, resulting very representative of the proteolytic peptides formed. In fact, matrix effects were tackled and determined by adding the standard in a matrix (chickpea flour) very similar to the samples to be analyzed. Moreover, in the present work, the method was applied not only to soy samples but also to real foods containing soybeans or soy-derived products and compared to the results obtained by the indirect determination of the trypsin inhibition (according to BS EN ISO 14902:2001).

## Materials and Methods

### Chemicals

Hydrochloric acid (37%, AnalaR NORMAPUR), sodium hydroxide (pellets, AnalaR NORMAPUR), acetonitrile (for UPLC/UHPLC instruments, HiPerSolv CHROMANORM), and methanol (for UPLC/UHPLC instruments, HiPerSolv CHROMANORM) were purchased from VWR International (Milan, Italy). Sodium phosphate dibasic (pure) was purchased from Carlo Erba (Cornaredo, Italy). Ammonium bicarbonate (≥99.5%) was purchased from Fluka Analytical (St. Gallen, Switzerland). Urea (for synthesis) was purchased from Merck Schuchardt (Hohenbrunn, Germany). TI from *Glycine max* (soybean, lyophilized powder, 13471 U/mg), trypsin from porcine pancreas (lyophilized powder, 1000–2000 BAEE units/mg solid), α-chymotrypsin from bovine pancreas (type II, lyophilized powder, >40 units/mg protein), DL-dithiothreitol (≥99% titration), iodoacetamide (crystalline), calcium chloride (anhydrous), formic acid (≥96%), and Nα-benzoyl-l-arginine 4-nitroanilide hydrochloride (>98%, TLC) were purchased from Sigma-Aldrich (St. Louis, MO, USA).

### Samples

The soybean flours of two different varieties (Energy and Namaste) were kindly provided by the Department of Agricultural, Food, Environmental and Animal Sciences of the University of Udine, Italy. Soy burgers, soy milk, soy protein milk, tofu, yofu, and chickpea flour were bought at the local supermarket. Soy burgers and tofu were analyzed both raw and cooked. The cooking was carried out in the oven at 105 °C for 20 min. All samples, except for meals, were freeze-dried before protein extraction and digestion, using a Lio 5P freeze drier (5 Pascal, Milan, Italy) at 0.2–0.4 mbar and temperature between −45 and −50 °C.

### Protein Extraction and Digestion

One gram of sample was extracted with 6 mL of an aqueous solution of urea (4 M), ammonium bicarbonate (0.1 M), and dithiothreitol (0.005 M), as previously reported.^39^ After 40 min of stirring with a vortex and reciprocating shaker (Stuart Scientific, Vernon Hills, IL, USA), the samples were centrifuged (5810R, Eppendorf, Hamburg, Germany) at 3220*g* for 5 min at 4 °C. One mL of the supernatant was alkylated by incubation at 50 °C with 25 μL of iodoacetamide (0.5 M) for 20 min. Then, the sample was diluted with 1.64 mL of ammonium bicarbonate (0.025 M) and added with 200 μL of trypsin (1 mg/mL, dissolved in HCl 0.001 M, pH 3) and 200 μL of chymotrypsin (1 mg/mL, dissolved in CaCl_2_ 0.002 M, HCl 0.001 M, pH 3). These amounts correspond to an enzyme concentration of 130.5 μg/mL. Digestion was carried out overnight (18 h) at 37 °C in an incubator with an orbital shaker (ES-20, Biosan, Riga, Latvia). At the end of the digestion, the enzymes were inactivated with 500 μL of 10% (V/V) formic acid (1 mL for soybean meal). Prior to UHPLC/ESI-MS/MS analysis, samples were centrifuged at 6708*g* at 4 °C for 10 min and then filtered through syringe filters (0.45 μm). All the samples were prepared and analyzed in duplicate.

### Calibration Curve Preparation

Chickpea flour was used as a blank matrix, to which increasing amounts of Kunitz type inhibitor standard were added. The standard was added before the extraction phase, so to be submitted exactly to all the steps of the analyte in the samples. Then, the spiked chickpea flour was submitted to the same extraction and digestion procedure of the samples. Standards at different concentrations were prepared, in the range from 5.31 × 10^–2^ to 2 × 10^–6^ mg/mL. All the standards were prepared and analyzed in duplicate.

### UHPLC/ESI-MS/MS Analysis

LC–MS analysis was performed as previously described.^40^ The Aeris PEPTIDE 1.7 μm XB-C18 column (100 Å, 150 × 2.1 mm; Phenomenex, Torrance, CA, USA) was used for the chromatographic analysis. Chromatographic separation was performed in a Dionex Ultimate 3000 UHPLC (Sunnyvale, CA, USA). The flow was set at 0.2 mL/min, the column temperature at 35 °C and the sample temperature at 11 °C; eluent A was water with 0.1% (v/v) of formic acid and 0.2% (v/v) of acetonitrile, eluent B was acetonitrile with 0.1% (v/v) formic acid and 0.2% of water. A gradient elution was performed based on the following parameters: 0–7 min 100% A, 7–50 min from 100% A to 50% A, 50–52.6 min 50% A, 52.6–53 min from 50% A to 0% A, 53–58.2 min 0% A, 58.2–59 min from 0% A to 100% A, and 59–72 min 100% A (total analysis time 72 min). The injection volume was 5 μL. Detection was achieved using a triple quadrupole mass spectrometer (TSQ Vantage, Thermo Fisher Scientific, Waltham, MA, USA) with the following parameters: solvent delay 0–7 min, acquisition 7–58.2 min, ionization type positive ions; spray voltage 3500 V, vaporizer temperature 250 °C; sheath gas pressure 22; and capillary temperature 250 °C. For the Q1MS scan mode, the acquisition range was set to 100–1500 *m*/*z*. For the product ion scan mode, the collision energy was applied according to the formula: CE = 0.034 × (*m*/*z*) + 3.314^41^ and the acquisition range for fragment detection was 100–1500 *m*/*z*. For the selected reaction monitoring method, the monitored transitions are 670.36 → 187.01; 697.43; 925.50. UHPLC/ESI-MS data were processed using Xcalibur software (Thermo Fisher Scientific, Waltham, MA, USA). The calculations were carried out using both the TIC trace (sum of the three monitored transitions), and the trace of the individual transitions, obtaining completely comparable results.

### Determination of Trypsin Inhibitory Activity in Soya Products

Determination of trypsin inhibitory activity (TIA) was performed as described in the BS EN ISO 14902:2001 (animal feeding stuffs). Briefly, TIs are extracted from the sample at pH 9.5 (sodium hydroxide solution, 0.01 M) by overnight incubation at 4 °C (1 g of sample in 50 mL of solution). Then, samples are opportunely diluted to obtain a trypsin inhibition percentage between 40 and 60%. The remaining trypsin activity is measured by adding benzoyl-l-arginine-*p*-nitroanilide (L-BAPA) as the substrate. Sample extract is then incubated with water, trypsin working solution and L-BAPA for 10 min at 37 °C. The reaction is stopped by the addition of acetic acid. The quantity of released *p*-nitroaniline is measured spectrophotometrically at 410 nm.

### Bioinformatic Tools

Uniprot (www.uniprot.org, last accessed 07/02/2023) was used to perform Basic Local Alignment Sequence Tool (BLAST) using UniProtKB reference proteomes + Swiss-Prot as target database. Parameters: Sequence type: Protein; Program: blastp; E-Threshold: 10; Matrix: Auto - BLOSUM62; Filter: None; Gapped: yes; Hits: 250; HSPs per hit: All.

Fragment Ion Calculator (http://db.systemsbiology.net/proteomicsToolkit/FragIonServlet.html, last accessed 07/02/2023) was used to generate the in silico MS/MS fragmentation pattern of the peptides. Cysteine residues were considered as carbamidomethylated (+57).

Statistical analysis was performed using IBM SPSS Statistics (V. 28.0.0.1): data normality of the KTI3 content was checked with both Kolmogorov–Smirnov and Shapiro–Wilk tests (*p* < 0, 01 for both, so the data distribution is not normal). The homogeneity of the variance was verified using the Levene test (*p* < 0.01, therefore the variance is not homogeneous). The presence of significant differences between the food samples tested was then verified with the Kruskal Wallis test with independent samples and pairwise comparison (*p* < 0.05).

## Results and Discussion

### Identification of the Peptide Marker

**Figure 1:**
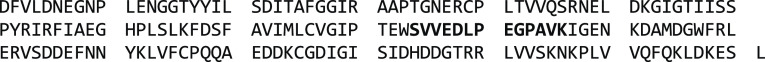
Amino acid sequence of for the Kunitz-type trypsin inhibitor KTI3 (Uniprot accession n. P01070). The marker peptide is reported in bold.

The first step of this work was the identification of a peptide marker for the Kunitz-type trypsin inhibitor KTI3 present in soybean, whose sequence (as reported in the Uniprot database for entry P01070) is reported in Figure 1.

To identify the marker peptide, preliminary experiments were performed on a pure standard of Kunitz-type inhibitor from *G. max*, commercially available. A first LC–MS analysis of the standard allowed the identification of the KTI 3 at Rt 45.67 min (Supporting Information, Figure S1). From the mass spectra, a MW of 20 092.77 Da can be calculated, consistent with the reported MW (20.1 kDa) of KTI3 (181 AA). A second major multicharged pattern can be identified under the same chromatographic peak, corresponding to the MW of 19 979.45 Da. The difference in MW (113.32 Da) can correspond to the loss of the C-term leucine, pre-analysis, or during ionization.

Criteria for the selection of the peptide marker were fixed as previously done:^40^

- Length about 8–10 amino acids (PM 800–1500).
- Specific cleavages of the enzyme used for digestion.
- Absence of missed cleavages.
- Absence of labile amino acids (e.g., cysteine).
- 100% match with the sequence of the protein of interest (KTI).

As a first approach, a classical digestion in solution was attempted using trypsin as the cleavage enzyme. However, enzymatic digestion did not prove to be exhaustive, as expected, as some intact protein was still detectable by LC–MS after tryptic digestion (Supporting Information, Figure S1). Probably, the reduction and alkylation steps (with dithiothreitol and iodoacetamide, respectively) were not sufficient to eliminate the inhibitory activity of this protein toward trypsin. The fact that the proteolytic reaction does not go to completion makes trypsin digestion unsuitable for the quantification purposes, as the remaining intact protein would make the result unreliable.

Then, a different enzyme (chymotrypsin) was tested for in-solution digestion. In this case, undigested protein was no longer detectable by either sodium dodecyl sulfate-polyacrylamide gel electrophoresis or LC–MS. Peptides were then identified by LC–MS/MS (Supporting Information, Table S1). All identified peptides were aligned in the protein database (UniprotKB) with the Basic Local Alignment Search Tool (BLAST) and only those specific to *G. max* were selected (Supporting Information, Table S2). Of these six peptides, none met all the requirements to become a marker peptide due to the presence of cysteine, missed cleavages, non-specific cleavages, or a mix of these conditions.

Finally, a combination of trypsin and chymotrypsin (1:1) was finally used to digest the Kunitz-type inhibitor KTI3. Different ratios of total enzyme to substrate were tested: 1:20, 1:10, and 1:5. The peptides resulting from the three conditions tested were comparable both in terms of sequence and quantity; therefore, the enzyme to substrate ratio of 1:20 was chosen for the experiments. After filtering the identified peptides with the fixed criteria, a marker peptide was finally identified: SVVEDLPEGPAVK (highlighted in bold in the protein sequence). Both variants P01070 and P01071 contain this peptide marker. N-term cleavage is specific for chymotrypsin (tryptophan at position P1), and C-term cleavage is specific for trypsin (lysine at position P1).

### Development of the LC–MS Method for the Quantification of the Kunitz-Type Trypsin Inhibitor KTI3

**Figure 2:**
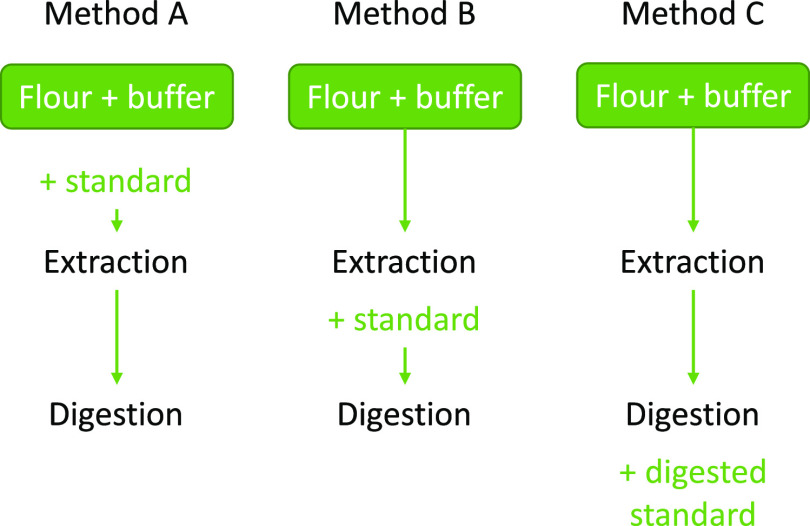
Three tested methods for preparing samples for calibration curves.

Once the peptide marker for the Kunitz-type trypsin inhibitor had been identified, the aim of the work was to develop a quantitative method for its determination in food products. As regards the construction of the calibration curve, chickpea flour was chosen as the blank matrix to be spiked with the standard at increasing concentrations since its composition is not much different from that of soy flour. To decide at which step of the procedure the standard should be added to the blank matrix, three different approaches were tested, as described in Figure 2:

(A) Blank flour + standard, followed by extraction, reduction, alkylation, digestion with trypsin and chymotrypsin, and LC–MS analysis.
(B) Extraction of the blank flour, followed by the addition of the standard, reduction, alkylation, digestion with trypsin and chymotrypsin, and LC–MS analysis.
(C) Extraction of the blank flour, reduction, alkylation, digestion with trypsin and chymotrypsin, addition of the standard (pre-digested separately with trypsin and chymotrypsin), and LC–MS analysis.

Furthermore, since different orders of magnitude of concentrations had to be covered, two different concentration ranges were tested, namely “low concentrations” (from 0.2 to 3.4 μg/mL) and “high concentrations” (from 3.0 to 53 μg/mL).

**Figure 3:**
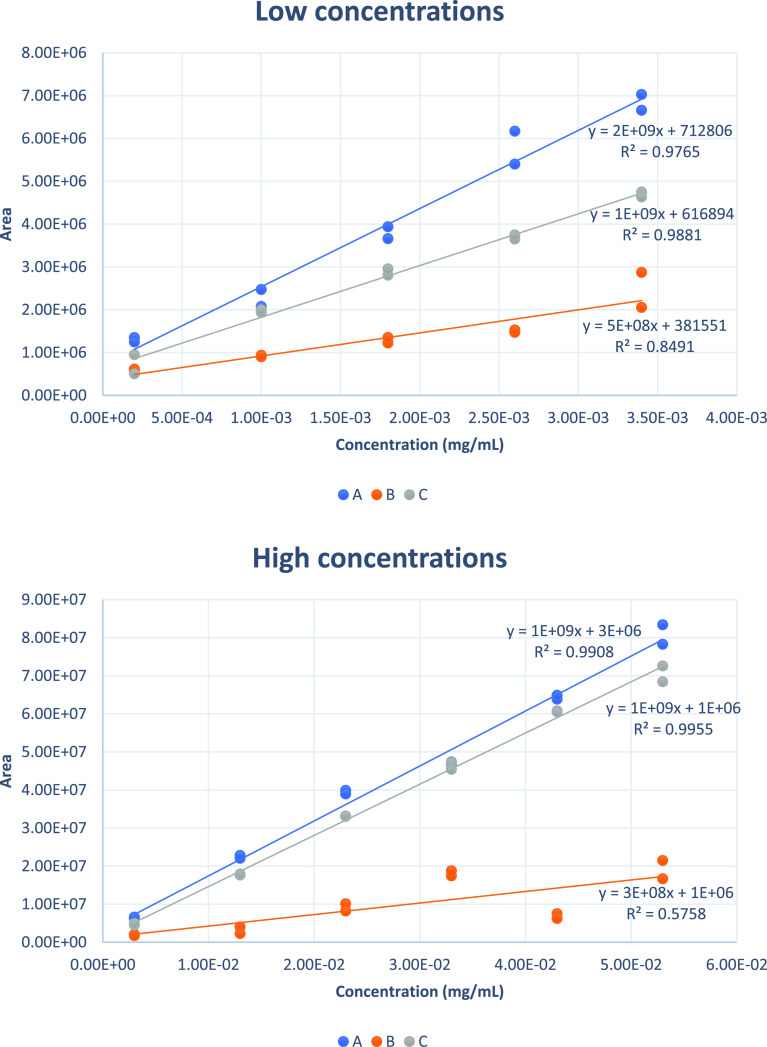
Calibration curves obtained by spiking the Kunitz-type trypsin inhibitor standard (at low and high concentrations) at different points in the procedure.

As can be seen (Figure 3), option B is always the worst, both in terms of linearity and sensitivity. In fact, the *R*^2^ values of option B are 0.8491 and 0.5758, very far from an optimal value close to 1, indicating poor linearity of the response. Also regarding sensitivity, the slopes of the calibration curve obtained with option B (3 × 10^8^ and 5 × 10^8^) are almost an order of magnitude lower than with options A and C, indicating a smaller variation of the response to equal change in concentration. As for options A and C, they are approximately equivalent at high concentrations, while at low concentration, option A has better sensitivity (slope of 2 × 10^9^ for option A and 1 × 10^9^ for option C), which is especially useful when working at low concentrations. The final protocol therefore was set up, including the addition of the standard to the blank flour at the very beginning of the procedure.

The limit of detection (LOD) and the limit of quantification (LOQ) of the method, calculated from the calibration curves (as reported in^19^) were, respectively, found to be (in terms of protein amount) 0.75 and 2.51 μg/g. These limits are lower than those previously obtained by LC-UV techniques^28,29^ and are comparable to KTI3 amount detected with LC–MS methods employing isotopically labeled peptides.^35−37^ The accuracy of the method was on average 93% (range 70–123%) for low concentrations (from 3.1 to 3.4 μg/mL) and 103% (range 86–115%) for high concentrations (13 to 53 μg/mL).

### Application of the Developed Method to Real Food Samples

To transfer the developed method to real food samples (rich in many other proteins than KTI3), the amount of enzymes was optimized by extracting and digesting two different soy varieties (Energy and Namaste). Three different enzyme concentrations were tested (65.2 μg/mL, 130.5 μg/mL, and 261 μg/mL), and the one giving the most intense chromatographic signals was 130.5 μg/mL, as reported in Supporting Information, Figure S2. Hence, this concentration was used for the analysis of all food samples tested. The developed method was applied for the quantification of the Kunitz-type trypsin inhibitor KTI3 in various food products containing soy or soy-derived proteins. In addition, two different soybean varieties (Namaste and Energy) were also analyzed. Results are reported in Table 1. KTI was detected and quantified in all samples analyzed, regardless of the type or cooking process.

**Table 1:** Concentrations of Kunitz-Type Trypsin Inhibitor KTI3 (mg/g) Determined by LC–MS in Various Food Products Containing Soy or Soy-Derived Proteins^a^

sample	protein content (%)	KTI3 (mg/g) determined by LC–MS	KTI3 (mg per medium portion)	TIA (mg/g) determined by UV
soybean (Energy)	39.3^b^	1.69 ± 0.22 (CV 13%)	143 (85 g)	>25.2
soybean (Namaste)	33.8^b^	0.01 ± 0.00 (CV 0%)	0.49 (85 g)	>25.2
soy drink	31.2^c^	8.15 ± 3.83 (CV 47%)	191 (244 g)	10.4
proteic soy drink	40.5^c^	79.14 ± 7.76 (CV 10%)	2387 (244 g)	8.3
Yofu	16.5^c^	17.21 ± 1.08 (CV 6%)	791 (245 g)	1.2
tofu (raw)	45.9^c^	14.84 ± 1.31 (CV 9%)	573 (124 g)	0.7
tofu (cooked)	48.0^d^	6.21 ± 4.95 (CV 80%)	230 (124 g)	0.6
soy burger (raw)	39.9^c^	9.00 ± 0.10 (CV 1%)	224 (71 g)	<0.5
soy burger (cooked)	42.1^d^	6.30 ± 0.06 (CV 1%)	149 (71 g)	<0.5

a As a comparison, the inhibitory activity on trypsin (TIA), determined spectrophotometrically, is also reported. All the results are expressed “as is” for the soybean flours, while for the other matrices, it is reported for the freeze-dried samples, so as not to be influenced by a strongly different water content. Average servings are calculated based on https://www.nutritionvalue.org/. CV: coefficient of variation (%). Statistically significant differences between pairs of samples for KTI3 (mg/g) determined by LC–MS: 2–6, 2–5, 2–4, 1–5, 1–4, 9–4, and 7–4.

b Determined by the Kjeldahl method.

c As indicated on the label.

d As indicated on the label, corrected for the water content after cooking.

As a first observation, all soybean products analyzed contain more KTI3 than raw soybean varieties. This probably indicates a concentration of KTI3 during the manufacturing process; another possible explanation could be better extractability of KTI3 in processed foods compared to crude matrices. In fact, all soy products undergo several processing steps during their production: soy drinks are produced by soaking and grinding soybeans, boiling the mixture, and filtering the remaining particles; yofu is produced by fermenting the soy drink from selected bacterial strains, while tofu is prepared from soy drink by coagulation, pressing the resulting tofu, pasteurization, and packaging. To make the soy burger, the raw materials are extruded, minced, mixed with the other ingredients, and pressed to form the burger. All these steps can have an impact on the extractability and bioactivity of the proteins, as can be seen from the results obtained.

Despite the comparable protein content, Namaste soy flour showed a lower content of Kunitz-type trypsin inhibitors than Energy soy flour. In fact, Namaste soybean flour was supplied to us as a “low antinutritional variety”. Thus, this indicates that different varieties might have very different contents of antinutritional factors; therefore, the agronomic selection of varieties with a reduced content of protease inhibitors could improve the nutritional properties of these proteins and plant foods. Another interesting application of the present method could be to identify the factors that have an impact on the Kunitz-type trypsin inhibitor KTI3 content in soybeans, such as the impact of climate, rainfall, fertilizing practices, and so on. As an example, for durum wheat α-amylase/TI CM3 (which is also an allergen), a significant effect of both wheat variety and growing area was demonstrated.^30,31^ For what concerns soy-based drinks, the content of KTI3 can be very different, up to 10-fold. In particular, the proteic soy drink has higher KTI3 content of the conventional one. The higher protein content of the protein drink can only partially explain this difference, while other factors probably play an important role; some hypotheses may be different soy flours used as starting raw material, or different production processes which can cause KTI3 degradation or concentration. Yofu (a kind of soy analogue of milk yogurt) also contains KTI3, indicating that the fermentation process is unable to (completely) destroy this type of antinutritional factor. For both tofu and soy burgers, the amount of KTI3 decreases after heat treatment, consistent with the general idea that antinutritional factors are inactivated during cooking. However, they are still detectable in good quantities even after the applied heat treatment. Considering the average portions of the foods analyzed, the greatest intake of KTI3 would derive from the consumption of the soy protein drink, while the consumption of Namaste soy flour would lead to a negligible intake of KTI3.

### Comparison of Direct and Indirect Methods

Finally, the inhibitory activity of the samples on trypsin was also measured with the available spectrophotometric method, and the results are reported in Table 1. First, it should be emphasized that, although both results (LC–MS and UV–vis) are expressed in mg/g, they represent conceptually very different entities. The LC–MS methods output the mg of Kunitz-type trypsin inhibitor KTI3 in 1 g of sample, while the UV–Vis method measures the mg of inhibited trypsin from each gram of sample. As can be seen from Table 1, there is no significant correlation between these two measures, as also verified by the correlations of Pearson, Tau_b of Kendall and Rho of Spearman (*p* < 0.05). Both soybean meals, despite having the lowest KTI3 content, have TIA above the upper limit of the spectrophotometric method, indicating the presence of many other trypsin inhibitory compounds in addition to KTI3 (such for example other KTIs, or the Bowman-Birk inhibitor, or others). The inhibitory activity on trypsin seemed to decrease with the increase in degree of processing of the products, and of course with the dilution of soy (or soy protein) with other ingredients. Indeed, TIA was found to be lower for soy beverages, even though the difference in KTI3 content was not reflected in the TIA, which was found to be similar between the two samples. In yofu and tofu, the TIA was found to be lower than in beverages, and again, the TIA was not related to the KTI3 content. The TIA was found to be not very different between raw and heat-treated products (tofu and burger), and this can be partially explained by two hypotheses: the soy or soy protein may have already been treated before being included in the final product, so the antinutritional compounds may have already been denatured in the raw products; the TIA values are close to the lower limit of the method, so differences between the samples may have been flattened. The values of TIA for soy-based products are consistent with those found previously.^42,43^

The method presented here has the advantage of being a direct method, which clearly and unambiguously identifies and quantifies the Kunitz-type inhibitor KTI3 in soybeans and derivatives products. This avoids interference from other trypsin-inhibiting compounds, which can affect the indirect method based on the inhibition of the trypsin activity measured by UV–vis spectrophotometry. The accurate quantification of the KTI can find various applications: identification of soybean varieties with a low content of antinutritional factors, agronomic practices aimed at decreasing the content of protease inhibitors, particular climatic factors impacting on the KTI3 content, with the general aim of produce soybean and derived products with better digestibility. However, the direct measurement of the molecule and not of its activity (as occurs instead for the indirect method) has the limitation that the KTI3 could be present in a denatured and inactive form, therefore detectable by the LC–MS method, but having totally or partially loose its inhibitory activity. The method is in fact efficient for monitoring the presence of antinutritional KTI; then, if it is present, the inhibitory activity can be subsequently tested.

## Supporting Information Available

Peptides identified by LC–MS/MS in the chymotryptic digest of the Kunitz-type inhibitor standard from *G. max,* specific peptides identified for soybean Kunitz-type inhibitor KTI3, and LC–MS chromatograms and mass spectra of undigested and digested Kunitz-type inhibitor standard from *G. max* (PDF)

## Abbreviations used

DAD: diode array detection
ELISA: enzyme linked immunosorbent assay
KTI: Kunitz type inhibitor
L-BAPA: benzoyl-l-arginine-*p*-nitroanilide
LC-MS: liquid chromatography–mass spectrometry
LOD: limit of detection
LOQ: limit of quantification
MS: mass spectrometry
PAGE: polyacrylamide gel electrophoresis
TI: trypsin inhibitor
UV: ultraviolet
