# Supplementary material for: Label-Free Quantification by Liquid Chromatography–Tandem Mass Spectrometry of the Kunitz Inhibitor of Trypsin KTI3 in Soy Products
Source: J Agric Food Chem. 2023 May 23;71(22):8648–55. doi: 10.1021/acs.jafc.3c01173 (PMC10251515; doi:10.1021/acs.jafc.3c01173)

Supporting information

Label-free quantification by liquid chromatography – tandem mass spectrometry of the Kunitz inhibitor of trypsin KTI3 in soy products

Barbara Prandi*, Chiara Vacca, Stefano Sforza, Tullia Tedeschi

Department of Food and Drug, University of Parma, parco area delle scienze 17/A, 43124 Parma, Italy

* Corresponding author: Barbara Prandi, barbara.prandi@unipr.it, +39 0521 90 5431

Table of contents

Table S1……….Page S3

Table S2……….Page S5

Figure S1……….Page S6

Figure S2……….Page S7

Table S1. Peptides identified by LC-MS/MS in the chymotryptic digest of the Kunitz-type inhibitor standard from *Glycine max*.

Rt	m/z	z (n)	MW	$MW_{average}$	Sequence	a	b	c	x	y	z	Charge	BLAST (100%)
13,85	**402,19**	2	802,38	802,44	TVVQSRN	173,15 (2)	201,08 (2)<br>671,20 (6)<br>785,37 (7)		401,98 (3)	376,08 (3)<br>504,39 (4)<br>603,30 (5)<br>702,42 (6)	359,07 (3)<br>586,5 (5)	1	25 proteins from different organisms, not specific
	803,50	1	802,50			201,08 (4)			201,08 (3)	302,23 (5)<br>401,98 (7)	293,13 (5)	2	
20,50	**416,75**	2	831,50	831,50	KLDKESL		128.84 (1)			218.58 (2)<br>591.40 (5)<br>704.17 (6)		1	127 proteins from different organisms, not specific
22,89	**593,22**	2	1184,44	1184,61	AAPTGNERCPL		143,03 (2)<br>512,97 (6)<br>797,29 (8)		255,45 (2)	228,73 (2)		1	P01070 (Trypsin inhibitor A, *Glycine max*); P01071 (Trypsin inhibitor B, *Glycine max*); Q39898 (Kunitz trypsin inhibitor, *Glycine max*); A0A0B2P2X3 (Trypsin inhibitor A, *Glycine soja*)
									407,82 (8)	322,68 (5)<br>407,82 (7)		2	
	1185,77	1	1184,77										
27,94	**670,22**	2	1338,44	1338,71	SVVEDLPEGPAVK	158,68 (2)<br>712,46 (7)	186,73 (2)<br>285,69 (3)<br>529,92 (5)<br>1193,72 (12)		343,81 (3)<br>1080,28 (10)	414,27 (4)<br>471,17 (5)<br>697,43 (7)<br>810,52 (8)<br>925,57 (9)<br>1054,47 (10)<br>1153,86 (11)	229,11 (2)<br>907,81 (9)	1	P01070 (Trypsin inhibitor A, *Glycine max*); P01071 (Trypsin inhibitor B, *Glycine max*); Q39898 (Kunitz trypsin inhibitor, *Glycine max*); A0A0B2P2X3 (Trypsin inhibitor A, *Glycine soja*)
	1339,98	1	1338,98							158,68 (3)<br>577,27 (11)<br>670,64 (13)		2	
30,09	**422,28**	2	842,56	842,45	SLKFDSF		201,01 (2)<br>591,47 (5)<br>678,48 (6)	695,98 (6)		253,11 (2)<br>643,41 (5)<br>756,60 (6)		1	127 proteins from different organisms, not specific
							165,52 (3)			422,42 (7)		2	
	843,33	1	842,33										
32,10	569,84	4	2275,36	2276,60									
	**759,96**	3	2276,88		TVVQSRNELDKGIGTIISSPY		201,08 (2)<br>1143,14 (10)<br>1270,61 (11)			278,71 (2)<br>365,72 (3)	991,1 (10)	1	P01070 (Trypsin inhibitor A, *Glycine max*); Q39898 (Kunitz trypsin inhibitor, *Glycine max*); A0A0B2P2X3 (Trypsin inhibitor A, *Glycine soja*)
						201,08 (4)	913,40 (17)<br>1000,83 (19)					2	
						434,53 (12)						3	
	1139,78	2	2277,56										

32,45	**746,87**	2	1491,74	1492,01	ELDKGIGTIISSPY		656,36 (6)<br>927,33 (9)<br>1040,80 (10)<br>1128,16 (11)<br>1215,31 (12)			278,92 (2)<br>365,86 (3)<br>1251,01 (12)		1	P01070 (Trypsin inhibitor A, Glycine max); Q39898 (Kunitz trypsin inhibitor, Glycine max); A0A0B2P2X3 (Trypsin inhibitor A, Glycine soja)
							656,36 (13)	365,86 (7)				2	
	1493,28	1	1492,28										
33,31	**691,78**	2	1381,56	1381,73	IGENKDAMDGWF		170,85 (2)<br>300,17 (3)<br>413,61 (4)<br>728,34 (7)<br>859,41 (8)<br>974,46 (9)<br>1031,60 (10)<br>1217,64 (11)			165,67 (1)<br>352,06 (2)<br>1083,71 (9)<br>1269,89 (11)	335,07 (2)	1	P01070 (Trypsin inhibitor A, Glycine max); Q39898 (Kunitz trypsin inhibitor, Glycine max); A0A0B2P2X3 (Trypsin inhibitor A, Glycine soja)
						595,25 (11)	609,30 (11)	159,03 (3)<br>618,33 (11)		691,90 (12)		2	
	1382,89	1	1381,89										
34,27	**879,45**	1	878,45	878,45	ILSDITAF	198,63 (2)<br>514,05 (5)<br>686,67 (7)	227,05 (2)<br>429,00 (4)<br>542,40 (5)<br>643,41 (6)<br>714,46 (7)<br>861,60 (8)			337,93 (3)<br>879,52 (8)			6 proteins from different organisms, not specific
37,90	521,75	2	1041,50	1041,53									
	**1042,55**	1	1041,55		YILSDITAF	135,84 (1)<br>249,38 (2)	277,17 (2)<br>390,43 (3)<br>592,52 (5)<br>705,15 (6)<br>806,09 (7)<br>877,21 (8)<br>1024,35 (9)		592,52 (5)	1042,76 (9)			P01070 (Trypsin inhibitor A, *Glycine max*); P01071 (Trypsin inhibitor B, *Glycine max*); Q39898 (Kunitz trypsin inhibitor, *Glycine max*); A0A0B2P2X3 (Trypsin inhibitor A, *Glycine soja*)

Table S2. Specific peptides identified for soybean Kunitz type inhibitor KTI3.

Rt	**m/z**	z	MW	Sequence	**BLAST (100%)**	Fragment 1	Fragment 2	Fragment 3	Defects
22,91	**593,22**	2	1184,44	(R)AAPTGNERC̲PL(T)	P01070 (Trypsin inhibitor A, *Glycine max*);<br>P01071 (Trypsin inhibitor B, *Glycine max*);<br>Q39898 (Kunitz trypsin inhibitor, *Glycine max*);<br>A0A0B2P2X3 (Trypsin inhibitor A, *Glycine soja*)	522,21	845,28	228,	Cysteine, nonspecific N-term
27,68	**670,22**	2	1338,44	(W)SVVEDLPEGPAVK(I)	P01070 (Trypsin inhibitor A, *Glycine max*);<br>P01071 (Trypsin inhibitor B, *Glycine max*);<br>Q39898 (Kunitz trypsin inhibitor, *Glycine max*);<br>A0A0B2P2X3 (Trypsin inhibitor A, *Glycine soja*)	697,36	186,87	158,75	Nonspecific C-term
31,97	**746,87**	2	1491,74	(N)ELDKGIGTIISSPY(R)	P01070 (Trypsin inhibitor A, Glycine max);<br>Q39898 (Kunitz trypsin inhibitor, Glycine max);<br>A0A0B2P2X3 (Trypsin inhibitor A, Glycine soja)	278,78	365,72	1214,89	Nonspecific N-term, 1 missed cleavage
32,14	**759,96**	3	2276,88	(L)TVVQSRNELDKGIGTIISSPY(R)	P01070 (Trypsin inhibitor A, *Glycine max*);<br>Q39898 (Kunitz trypsin inhibitor, *Glycine max*);<br>A0A0B2P2X3 (Trypsin inhibitor A, *Glycine soja*)	278,85	1000,41	365,51	1 missed cleavage
33,31	**691,78**	2	1381,56	(K)IGENKDAMDGWF(R)	P01070 (Trypsin inhibitor A, Glycine max);<br>Q39898 (Kunitz trypsin inhibitor, Glycine max);<br>A0A0B2P2X3 (Trypsin inhibitor A, Glycine soja)	158,96	595,18	1031,6	1 nonspecific cleavage, 2 missed cleavages
37,64	**1042,55**	1	1041,55	(Y)YILSDITAF(G)	P01070 (Trypsin inhibitor A, *Glycine max*);<br>P01071 (Trypsin inhibitor B, *Glycine max*);<br>Q39898 (Kunitz trypsin inhibitor, *Glycine max*);<br>A0A0B2P2X3 (Trypsin inhibitor A, *Glycine soja*)	592,38	705,36	277,03	2 missed cleavages

Figure S1. LC-MS chromatograms and mass spectra of undigested and digested Kunitz-type inhibitor standard from *Glycine max*.

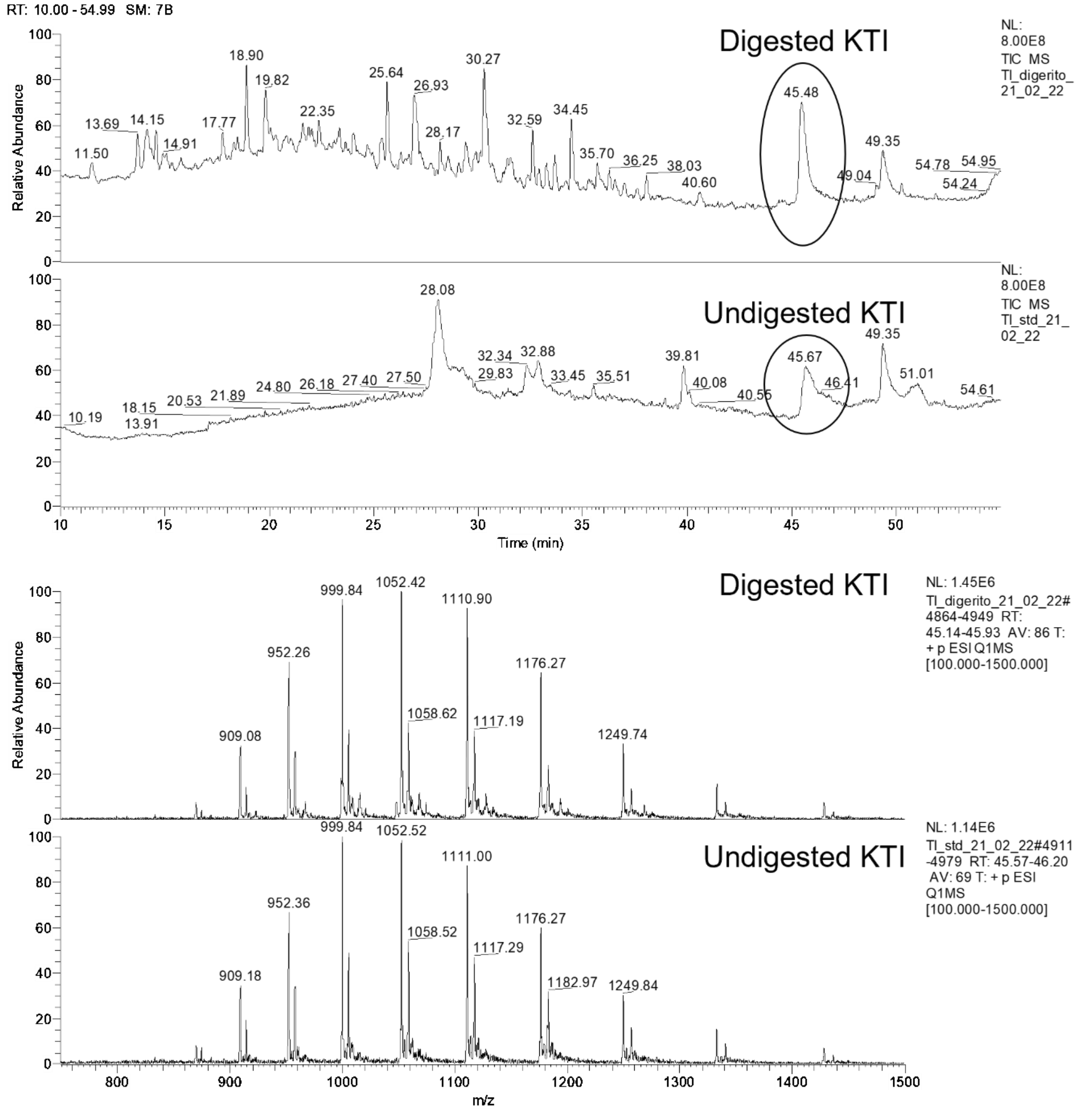

Figure S2. SRM chromatograms obtained from the analysis of Energy flour (E1, on the left) and Namaste flour (N1, on the right). 25, 50, and 100 represent a total enzyme concentration during digestion of 65.2 µg/mL, 130.5 µg/mL, and 261 µg/mL, respectively.

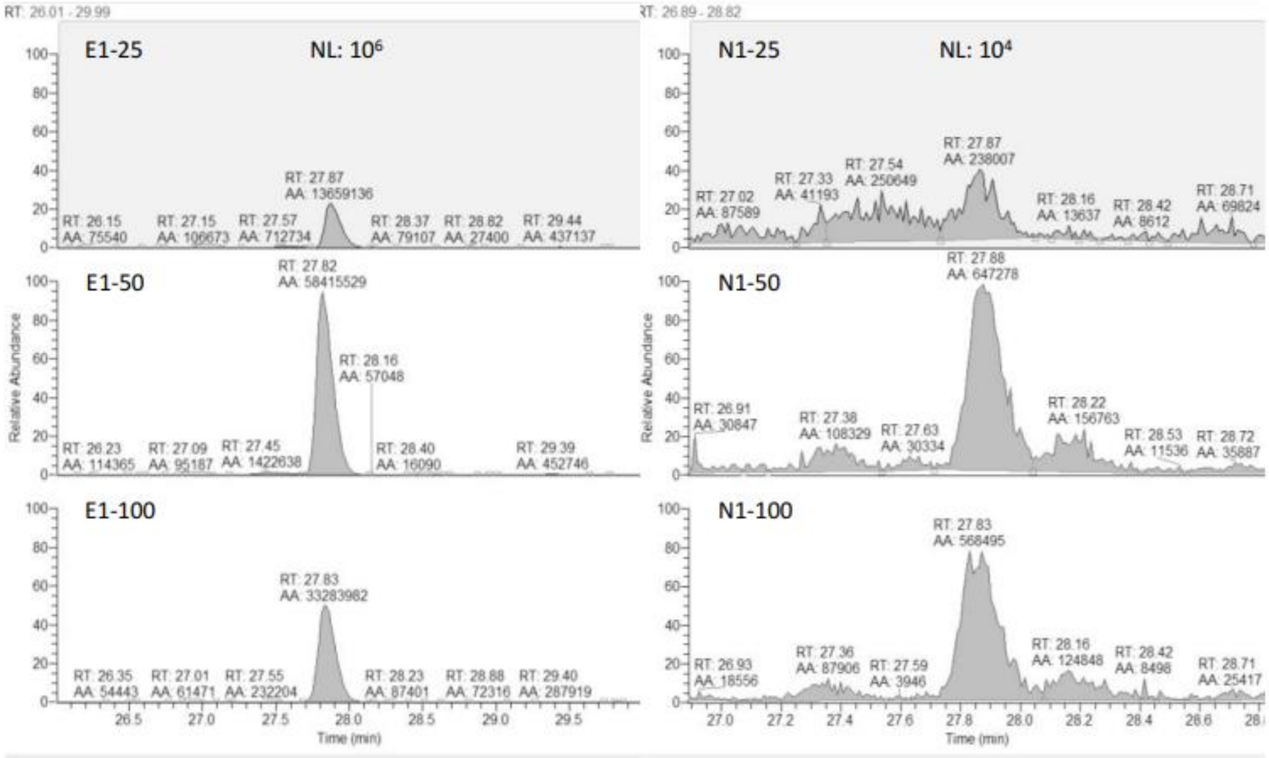

Supplement: Supplementary file 1 — jf3c01173_si_001.pdf [file jf3c01173_si_001.pdf]
